# Evaluation of the genes *CYP*_*85*_*A*_*2*_, *BZR1*, and *CAD1* in the attenuation of cadmium in seedlings of the species *Schizolobium parahyba *var.* amazonicum* (*Huber ex Ducke*)* Barneby* (PARICÁ) under different concentrations of 24-epibrassinolide

**DOI:** 10.1007/s11356-026-37919-1

**Published:** 2026-06-23

**Authors:** Erick dos Santos Ribeiro, Cândido Ferreira de Oliveira Neto, Ednaldo da Silva Filho, Igor Guerreiro Hamoy, Ana Ecídia de Araújo Brito, Dênmora Gomes de Araújo, Juliana Freitas do Nascimento, Evelyn Luane Pinheiro de Figueiredo, Dayane dos Santos Costa, Lilian Tatiana Costa Barros, Vinícius Oliveira Amancio

**Affiliations:** 1 Present Address: Laboratório do Estudo da Biodiversidade em Plantas Superiores, University Federal Rural da Amazônia, Instituto de Ciências Agrárias, Avenida Tancredo Neves, no., Cep: 66.077-830, Belém, Pará, 2501 Brazil; 2 Laboratório de Sorologia E Biologia Molecular (LSBM), University Federal Rural da Amazônia, Avenida Tancredo Neves, no., Cep: 66.077-830, Belém, Pará, 2501 Brazil; 3 Laboratório de Genética Aplicada (LGA), University Federal Rural da Amazônia, Avenida Tancredo Neves, no. , Cep: 66.077-830, Belém, Pará, 2501 Brazil; 4 Laboratório de Sementes (LABSEM), University Federal Rural da Amazônia, Avenida Tancredo Neves, no. , Cep: 66.077-830, Belém, Pará, 2501 Brazil

**Keywords:** Phytotoxicity, Molecular biology, Plant hormone, 24-epiBL, Bioregulation

## Abstract

The Amazonian species paricá (*Schizolobium parahyba *var.* amazonicum* (*Huber ex Ducke*) *Barneby*) accumulates cadmium (Cd) primarily in the roots, but the lack of understanding of gene modulation in response to this metal and the phytohormonal mechanisms complicates its relevance in the rehabilitation of degraded areas. Therefore, the present study aims to examine the expression of the *CYP*_*85*_*A*_*2*_ and *BZR1* genes, precursors of 24-EBL, in the production of chelating proteins by the *CAD1* gene in seedlings of the Paricá species, under various concentrations of CdCl_2_ and 24-EBL. The experiment was conducted in a growth room at the Laboratory of Studies on Biodiversity of Higher Plants (EBPS) at the Federal Rural University of Amazon (UFRA), Belém-Pará Campus, following a completely randomized experimental design (CRD), in a 4 × 3 factorial scheme, totaling 60 experimental units with four treatments of CdCl_2_ (0, 50, 100, and 150 µM) and three doses of 24-epibrassinolide (0, 20, and 40 nM). The data were subjected to analysis of variance (ANOVA) (*p* < 0.05), and the differences between treatments were analyzed using Tukey’s test (*p* < 0.05). The biometric variables indicated a significant reduction in root length due to the harmful effect of CdCl_2_. Additionally, losses in *Chl a*, *Chl b*, and *Chl a* + *b* were observed due to the entry of Cd^2+^ into the leaf tissues. Despite this, the *CYP*_*85*_*A*_*2*_, *BZR1*, and *CAD1* genes showed greater expression in the aerial part with varying doses of 24-EBL, while *CAR* and *ACN* were affected by increased CdCl_2_, indicating a genetic adjustment in the upper parts of the plants to cope with Cd toxicity and maintain biological functions.

## Introduction

Anthropic action, resulting from activities such as uncontrolled mining and industrialization, leads to the introduction of heavy metals into the environment (Bendito et al. 2017; Benavente et al. 2020). These heavy metals, including mercury (Hg), arsenic (As), and cadmium (Cd), are released into the soil and water in alarming quantities, polluting terrestrial and aquatic ecosystems (Silva & Lima 2017; Shi 2019; Ali et al. 2019; Balali-Mood et al. 2021).

Cadmium, in particular, is a phytotoxic metal easily absorbed by plants, posing a threat to ecosystems due to its entry into the food chain (Fernandes & Mainier 2014; Kubier et al 2019). Even at low concentrations, its absorption through roots negatively affects mineral nutrition and plant growth (Pan, 2010; Lin, 2015; Sun et al. 2021). Its high solubility in water facilitates its uptake by plants, compromising their development and homeostasis (Sousa, 2018; Bastos et al. 2021). Visible symptoms include leaf curling and yellowing, impacting water absorption and stomatal function (Souza et al. 2022; Rybarczyk et al. 2025). It damages the photosynthetic system, reducing chlorophyll and carotenoids, decreasing photosynthetic efficiency, and inhibiting CO_2_ fixation enzymes (Tabelin et al. 2018; Moravcíkova & Ziarovská, 2023). In several plant species, cadmium toxicity can lead to genetic anomalies and disturbances in cell division (Gu et al. 2021).

In response to environmental challenges, plants have developed complex cellular communication systems, known as hormones, enabling rapid responses to adverse conditions (Bucker et al. 2017; Guo et al. 2019; Hu et al. 2021). Current studies focus on brassinosteroids (BR) and 24-epibrassinolide (24-EBL), which influence gene expression and metabolism, affecting growth and cell differentiation (Tong & Chu 2018; Zheng et al. 2022; Wahab et al. 2022). Genes, essential to genetic inheritance, are DNA segments that define traits and ensure the continuity and diversity of species (Moore et al. 2021). This genetic information is crucial for cellular function and phenotype expression (Sabella et al. 2021).

The *BZR1* and *CPY*_*85*_*A*_*2*_ genes mediate the action of 24-EBL, playing a fundamental role in 24-EBL production in response to plant exposure to Cd (Rajewska et al. 2016; Wei and Li 2020). BZR1 is a key regulator in the BR signaling cascade, including 24-EBL, while CPY85A2 is involved in converting plant steroids into bioactive forms like 24-EBL (Zullo & Adam 2002; Rahmani et al. 2021). One of the primary effects of 24-EBL is stimulating the production of phytochelatin proteins derived from the CAD1 gene, which bind to Cd, reducing its toxicity in plant cells (Ren et al. 2023).

Additionally, 24-EBL induces the expression of antioxidant enzymes that help neutralize free radicals produced in response to Cd (Luo & Zhang 2021). However, the lack of studies on genetic regulation related to cytological responses to Cd and 24-EBL hinders the understanding of its role in cellular stability in plants (Bilal et al. 2023). In the Amazon, research seeks pollutant-resistant plants for reforestation projects (Eras et al. 2022). The Paricá (*Schizolobium parahyba *var.* amazonicum*) stands out for its rapid growth, making it a valuable option for reforestation with economic and environmental potential (Rodrigues et al. 2018). Its fast growth makes it attractive to cooperatives and timber industries in the short term (Oliveira et al. 2023). In the presence of Cd, variations occur in the amount accumulated in different organs (leaves and roots), impacting nitrogen (N) uptake and proline production, which acts as an osmotic regulator to reduce biological damage (Bastos et al. 2023). Although Cd concentration is more evident in roots, understanding genetic traits is crucial for comprehending this species’ resistance to the metal.

Thus, the present study aims to analyze the expression of the cytochrome P450 (*CYP*_*85*_*A*_*2*_), brassinazole-resistant 1 (*BZR1*), and phytochelatin synthase 1 (*CAD1*) genes during the exogenous application of the phytohormone 24-epibrassinolide in the species Paricá (*Schizolobium parahyba *var.* amazonicum* (*Huber ex Ducke*) *Barneby*) subjected to oxidative stress under different dosages of CdCl_2_.

## Material and methods

The experiment was conducted in the growth room at the Laboratory for the Study of Biodiversity in Higher Plants (EBPS), located at the Institute of Agricultural Sciences (ICA) of the Federal Rural University of the Amazon (UFRA), Belém-PA Campus. The biochemical analyses were carried out at the Biochemistry Laboratory of EBPS. The seeds, totaling 600, were provided by the Seed Laboratory (LABSEM) of UFRA, Belém Campus. In the initial stages of seedling production for Paricá (*Schizolobium parahyba *var.* amazonicum* (*Huber ex Ducke*) *Barneby*), the seeds were scarified using 80-grit sandpaper and soaked in a solution containing 24-EBL (24-epibrassinolide) and deionized water (control) for 24 h to aid in breaking dormancy. Subsequently, the seeds were sown in pots with washed sand substrate, sterilized in an autoclave, and dried in an oven at 80 °C.

### Experimental design

The experimental design was completely randomized (CRD) in a 4 × 3 factorial scheme, with 12 treatments divided among four doses of cadmium chloride (0 µM, 50 µM, 100 µM, and 150 µM) and three levels of brassinosteroids in the form of 24-epibrassinolide (0, 20, and 40 nM of EBL), with five replications, each pot containing 15 plants per pot.

### Biometrics

Seedling height and root length were measured at the end of the experiment, on the 14th day, using a centimeter ruler. Biometric measurements were taken from the base of the stem to the apical bud of the plants.

### Determination of chlorophyll a, b, total (a + b), carotenoids, and anthocyanin levels

To obtain the total chlorophyll content, the simple calculation was used: Total Chlorophyll = Chlorophyll *a* + Chlorophyll *b*. First, 100 mg of fresh leaf from each treatment was weighed and placed in a crucible with ice for sample maceration, using 3 mL of 80% acetone. After maceration, the samples were centrifuged, and the supernatant was transferred to a 25-mL volumetric flask, with the volume adjusted using 80% acetone. The samples were then read in a spectrophotometer at 663 nm (chlorophyll *a*), 647 nm (chlorophyll *b*), 537 nm (carotenoids), and 470 nm (anthocyanins), with a blank (80% acetone) used to zero the device beforehand.

### Gene expression

To begin the molecular analyses, total RNA extraction was performed from the seedlings in the EBPS Laboratory, located at the UFRA Campus. First, samples of seedlings subjected to the treatments (CdCl₂ × 24-EBL) and the control were separated. A total of 5 mg of fresh leaflets and roots were separated and placed in Eppendorf tubes containing 2 mL of a homemade RNAlater solution for subsequent storage in an ultrafreezer at − 80 °C until RNA extraction could begin.

### Quantification and purity of RNA samples

The assessment of RNA purity and quantification was conducted at the Applied Genetics Laboratory (LGA), located on the UFRA Campus, using the Biodrop Duo UV/Vis spectrophotometer. The purity of the samples was then calculated through the ratio of absorbance measured at 260 and 280 nm (260/280 ratio), with results close to 1.8 considered to have an acceptable degree of purity. Subsequently, the samples were diluted, adjusted to a concentration of 50 ng/μL, and treated with DNase I, RNase-free (Thermo Scientific), following the manufacturer’s instructions.

### Real-time qPCR molecular analysis

The qRT-PCR analyses were performed using the one-step method at the Serology and Molecular Biology Laboratory, UFRA Campus, Belém, PA. The samples were standardized in duplicate alongside the genes described in Table 1. For single-product amplification, the samples were adjusted to a final volume of 10 μL, containing 1 × Power SYBR® Green RNA-to-CT™ One-Step Kit (Applied Biosystems, Foster City, CA, USA), 0.03 μL of reverse transcriptase, 4.4 μL of ultrapure water, 1 μL of RNA, and 0.36 μL of forward and reverse primer pairs (Table 1). All reactions were carried out in the CFX96 Touch™ Real-Time Detection System thermocycler (Bio-Rad, Hercules, CA, USA) following the protocol recommended by the kit manufacturer. Gene expression was estimated using the 2^−ΔΔCT^ method, with the actin gene used as a reference to stabilize the target genes in this study.

**Table 1:** Oligonucleotide sequences for gene expression analysis in the leaves and roots of *S. parahyba *var.* amazonicum*, using primer sequences with the qRT-PCR technique

Gene	Primers	GenBank
*CPY*_*85*_*A*_*2*_	F AAGAATGCTCGTCGTCCTCC R ATCTCCTCTGGCACCCATCT	ID: 816,394
*CAD1*	F CAGGGGTAGAGAACTGGGGA R GGATGGTGTGAGACCTAGCG	ID: 834,430
*BZR1*	F TCTCAACTCCGTTCCGTTTC R TGACGAAGAAGCCACAACTG	ID: 843,845
*ACTIN*	F GAAGCACCTCTCAACCCCAA R GGAAAGGACCGCCTGGATAG	ID: 14,763,233

*CYP*_*85*_*A*_*2*_ cytochrome *P450*, *CAD1* phytochelatin, *BZR1* protein of the brassinosteroid signaling positive regulator family, *ACTIN* actin.

### Experimental statistics

The biometric, biochemical, and molecular variables obtained were subjected to statistical analysis using analysis of variance (ANOVA) in the R software, version 4.2.1. The means were compared using the Tukey test at a 5% probability level. Graphs were created using R Studio software, version 1.3.1093, with the ggplot2 package and the “rcolorbrewer” function.

## Results

### Influence of CdCl_2_ and 24-EBL on biometric parameters in the species* S. parahyba *var.* amazonicum (Huber ex Ducke) Barneby (Paricá)*

**Fig. 1:**
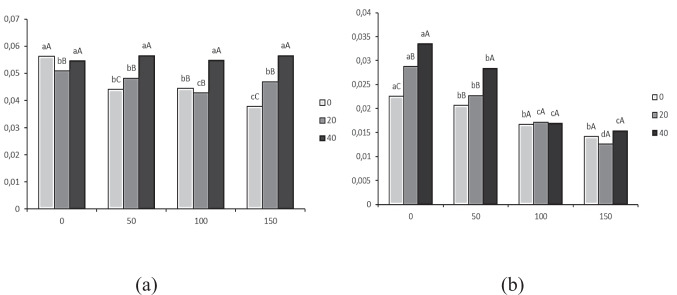
Effect of CdCl_2_ (0 µM, 50 µM, 100 µM, and 150 µM) and 2-EBL (0 nM, 20 nM, and 40 nM) on seedling height (**a**) and root length (**b**) of the species *Schizolobium parahyba *var.* amazonicum (Huber ex Ducke) Barneby* (PARICÁ). Lowercase letters indicate statistical differences among CdCl_2_ treatments (*p* < 0.05) based on Tukey’s test; uppercase letters indicate statistical differences among 24-EBL treatments (*p* < 0.05) based on Tukey’s test

In Fig. 1a, regarding the aerial structure of Paricá seedlings under the coordination of phytohormonal attenuators (0, 20, and 40 nM of EBL) at various doses of CdCl_2_ (0 µM, 50 µM, 100 µM, and 150 µM), no significant difference (*p* < 0.05) was observed only in seedlings treated with 40 nM, which maintained growth stability. The percentages of loss and gain (PRA%) support this statement, showing minimal changes in plant growth when compared to the control (34% > 33% > 33% > 31%). However, the toxic effects of Cd were observed with greater severity in plant groups subjected to 0 and 20 nM of 24-EBL (Fig. 1a) at varying doses of CdCl_2_ (0 µM, 50 µM, 100 µM, and 150 µM), showing a statistical difference (*p* < 0.05). The PRA% rates of plants at doses of 0 (0.5% > − 21% > − 21.6% > − 33%) and 20 nM (9.5% > − 14.5% > − 23.6% > − 16%) EBL, corresponding to height variables, decreased as CdCl_2_ concentrations increased.

Regarding the root structures (Fig. 1b), a decline in root dimensions was observed. Despite the administration of 24-EBL (0, 20, and 40 nM), there was a reduction in root length at different concentrations of CdCl_2_ (0 µM, 50 µM, 100 µM, and 150 µM), with a statistically significant variation (*p* < 0.05) compared to the control group. This results in a reduction in root impairment due to the harmful effects of Cd ions.

### Influence of CdCl_2_ and 24-EBL on the photosynthetic pigments of the species* S. parahyba *var.* amazonicum (Huber ex Ducke) Barneby (Paricá)*

**Fig. 2:**
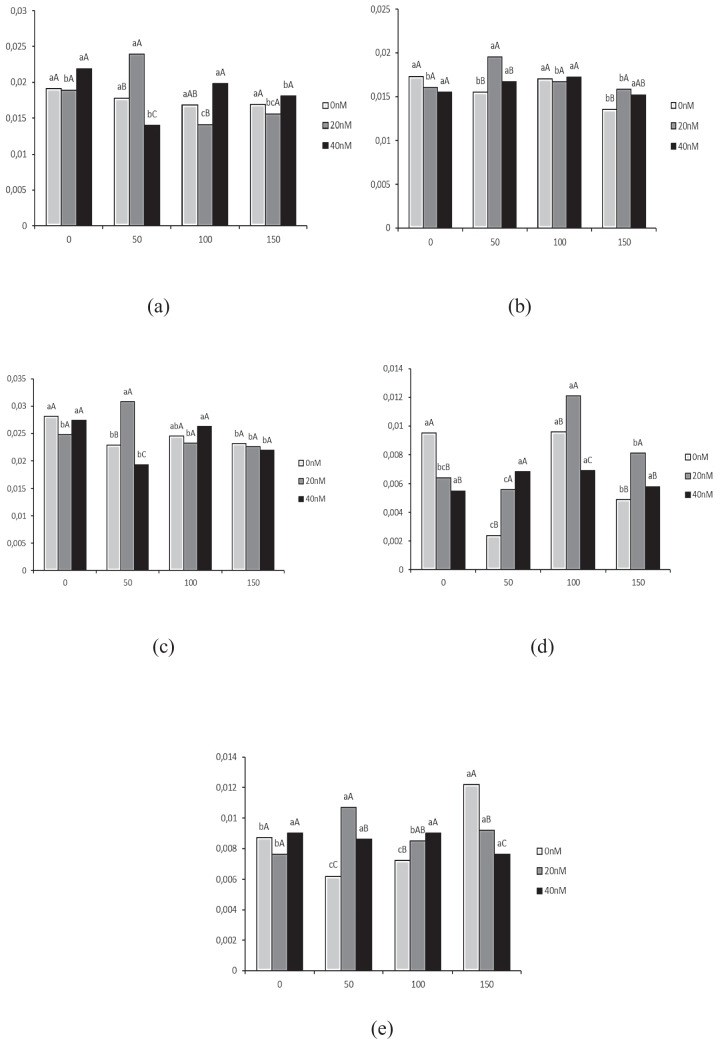
Influence of 24-epibrassinolide on the attenuation of CdCl_2_ in the distribution of chlorophyll a (**a**), chlorophyll b (**b**), total chlorophyll (a + b) (**c**), anthocyanins (**d**), and carotenoids (**e**) in the species *Schizolobium parahyba* var. *amazonicum* (PARICÁ). Lowercase letters indicate statistical differences among CdCl2 treatments (*p* < 0.05) based on Tukey’s test; uppercase letters indicate statistical differences among 24-EBL treatments (*p* < 0.05) based on Tukey’s test

The deleterious effects of Cd ions on photosynthetic pigments acted differently among the treatments. For samples treated with 50 µM of CdCl₂, a significant negative difference (*p* < 0.05) was observed in chlorophyll a (Fig. 2a) in the 40 nM 24-EBL segment. However, in chlorophyll b (Fig. 2b), the effect occurred at 0 nM of EBL, directly affecting the total chlorophyll pigments (Fig. 2c), with a significant negative impact (*p* < 0.05) under the same CdCl₂ conditions (50 µM) between 0 and 40 nM of 24-EBL.

The dosage of 20 nM of 24-EBL under the action of 50 µM of CdCl₂ showed a significant difference (*p* < 0.05) when compared to the control in Chl a (Fig. 2a), Chl b (Fig. 2b), and Chl a + b (Fig. 2c). Positive PRA% values were observed in the chlorophyll classes: Chl a (25.1%), Chl b (12.7%), and Chl a + b (9.6%).

The extracts from samples corresponding to the 100 and 150 µM CdCl₂ indices reveal a statistically significant negative variation (*p* < 0.05) in the 100 µM class under the interaction with 20 nM EBL in Chl a (Fig. 2a), b (Fig. 2b), and a + b (Fig. 2c). However, the highest degree of hazard was observed in the 150-µM segment, regardless of the 24-EBL phytohormonal class (0, 20, and 40 nM). Statistically significant differences (*p* < 0.05) were detected across all segments of Chl a, b, and a + b, which may have impaired the photosynthetic system of the species *S. parahyba *var.* amazonicum*.

Despite the changes in chlorophyll indices due to the severe toxic effects of CdCl₂, during the experiment, modifications were observed in the metabolism of antioxidant pigments such as carotenoids (CAR) and anthocyanins (ACN). Specifically, CAR pigments (Fig. 2e) increased significantly (*p* < 0.05) in plants subjected to 0, 20, and 40 nM of 24-EBL compared to variations in CdCl_2_ (0, 50, 100, and 150 µM). It is noteworthy that when exposed to 150 µM of CdCl_2_ and treated with 24-EBL (0, 20, and 40 nM), the plants recorded changes in PRA% of 40%, 15.7%, and 12.6%, respectively. Regarding ACN (Fig. 2d), a significant difference was observed in the stress response levels induced by the metal, according to the Tukey test (*p* < 0.05), particularly at concentrations of 50 and 150 µM of CdCl_2_ combined with 0 and 20 nM of 24-EBL. This contributed to the neutralization of reactive oxygen species in plant cells.

### Influence of Cd and 24-EBL on the CYP_85_A_2_ and BZR1 genes in the leaves and roots of the species* S. parahyba *var.* amazonicum (Huber ex Ducke) Barneby (Paricá)*

**Fig. 3:**
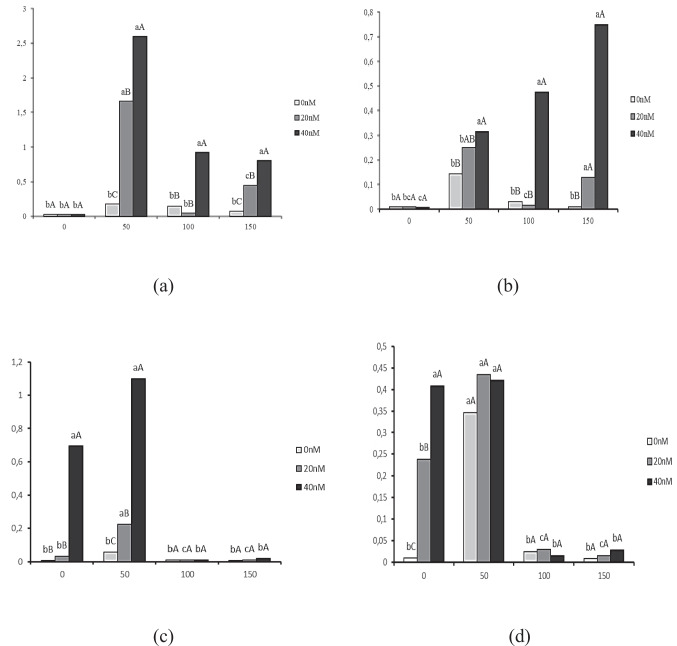
qPCR-RT analysis of the CPY_85_A_2_ and BZR1 genes of *Schizolobium parahyba *var.* amazonicum *(*Huber ex Ducke*)* Barneby* (PARICÁ) under different dosages of CdCl_2_ and 24-EBL. **a** qPCR-RT of the *CPY*_*85*_*A*_*2*_ gene located in the leaflets; **b** qPCR-RT of the *BZR1* gene located in the leaflets; **c** qPCR-RT of the *CPY*_*85*_*A*_*2*_ gene located in the root system; **d** qPCR-RT of the *BZR1* gene located in the root system. Lowercase letters indicate statistical differences between CdCl_2_ treatments (*p* < 0.05) based on the Tukey test; uppercase letters indicate statistical differences between 24-EBL treatments (*p* < 0.05) based on the Tukey test.

The RT-PCR evaluation of the seedlings’ leaflets was conducted after exposure to various concentrations of CdCl₂ (0, 50, 100, and 150 µM), influenced by different amounts of 24-EBL (0, 20, and 40 nM). A significant expression of the *CPY*_*85*_*A*_*2*_ (Fig. 3a) and *BZR1* (Fig. 3b) genes was observed, showing a notable statistical difference (*p* < 0.05) in the presence of 40 nM of 24-EBL. Interestingly, as the concentrations of CdCl₂ (50, 100, and 150 µM) increased, the PRA% of *CPY*_*85*_*A*_*2*_ decreased to 471%, 167%, and 146%, while for *BZR1*, the opposite occurred, with increases of 230%, 446%, and 716%.

Particularly in the root samples, a notable statistical similarity (*p* < 0.05) was observed between the *CPY*_*85*_*A*_*2*_ (Fig. 3c) and *BZR1* (Fig. 3 d) genes, precursors of EBL, when exposed to 40 nM of 24-EBL in the absence of CdCl_2_. However, upon introducing 50 µM of CdCl_2_, the first signs of increased expressivity were observed, with a statistically significant difference (*p* < 0.05) in plants under various doses of 24-EBL (0, 20, and 40 nM) for both *CPY*_*85*_*A*_*2*_ (Fig. 3c) and *BZR1* (Fig. 3 d) genes.

However, with the increase in concentrations to 100 and 150 µM of CdCl_2_ in the roots, a low expression of the *CPY*_*85*_*A*_*2*_ (Fig. 3c) and *BZR1* (Fig. 3 d) genes is observed in the root structures, with no statistical difference (*p* < 0.05) between 0 and 40 nM of EBL. At the 20 nM level of 24-EBL, a significant negative difference (*p* < 0.05) is observed, indicating the initial harmful effects of Cd on root tissues.

In summary, even at high concentrations of 50, 100, and 150 µM of CdCl_2_, the predominant genetic regulation occurred in the upper parts of the plants. In the roots, there was a reduction in gene stimuli, considering that they are the first organs to come into contact with toxic Cd ions. The application of 40 nM of 24-EBL stood out, emphasizing the importance of the *CPY*_*85*_*A*_*2*_ and *BZR1* genes, which are crucial in the 24-EBL metabolism in the leaves. This pattern suggests a unique response to CdCl_2_ exposure, highlighting the crucial role of the aerial part in gene regulation under stress conditions. These findings deepen the understanding of the molecular mechanisms related to the plant’s response to metal ions and growth-regulating substances.

### Influence of CdCl₂ and 24-EBL on the CAD1 gene in the aerial part and root of the species* S. parahyba *var.* amazonicum (Huber ex Ducke) Barneby (PARICÁ)*

**Fig. 4:**
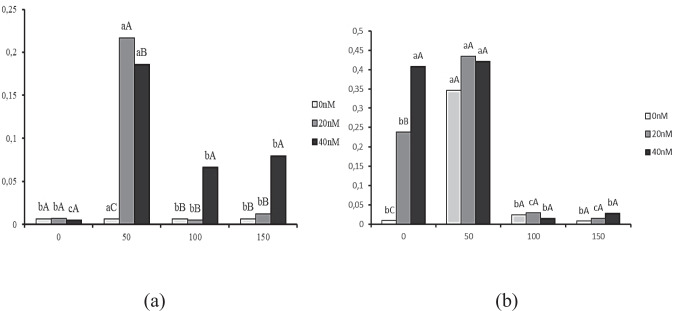
qPCR-RT analysis of the *CAD1* gene in the species *Schizolobium parahyba *var.* amazonicum *(*Huber ex Ducke*)* Barneby* (PARICÁ) under different dosages of CdCl_2_ and 24-EBL. **a** qPCR-RT of the *CAD1* gene located in the leaflets; **b** qPCR-RT of the *CAD1* gene located in the root system. Lowercase letters indicate statistical differences among CdCl_2_ treatments (*p* < 0.05) based on Tukey’s test; uppercase letters indicate statistical differences among 24-EBL treatments (*p* < 0.05) based on Tukey’s test.

The molecular analyses of *CAD1* levels in genetic material extracted from leaves and roots under various doses of phytoregulators (0 nM, 20 nM, and 40 nM) at 0 µM CdCl_2_ showed a significant difference (*p* < 0.05) only in the roots, with a progressive yield of PRA% (0% > 137% > 278%). However, the addition of the initial doses of Cd showed a statistically significant difference at the 5% level at 50 µM CdCl_2_ in the fractionation of 24-EBL (0 nM, 20 nM, 40 nM), with positive *CAD1* activity observed in both roots (Fig. 4b) and leaflets (Fig. 4a) during the experimental period.

Distinctly, in the root samples (Fig. 4b) treated with 100 µM and 150 µM of CdCl_2_, regardless of the attenuator segments used in 24-EBL (0 nM, 20 nM, 40 nM), no significant difference (*p* < 0.05) was observed when compared to the control, revealing early signs of biological instability. This impact was reflected in the leaves (Fig. 4a), showing no significant difference at the 5% level according to Tukey’s test in the 24-EBL applications.

## Discussion

### Modification in the growth of *S. parahyba var. amazonicum (Huber ex Ducke) Barneby (PARICÁ)* seedlings under different concentrations of CdCl2 and 24-EBL

In the aerial part, high concentrations of Cd trigger a series of events that generally reduce the growth of the main stems, redirecting energy investment toward lateral and root growth (Kim et al. 2010; Zhu et al. 2021). This adjustment is mediated by the inhibition of auxin activity, a plant hormone that promotes cell elongation (Bajguz et al. 2019; Groszyk and Hebda 2021; Qiao et al. 2022). Additionally, plants can increase the synthesis of antioxidant compounds, such as glutathione, to mitigate damage caused by Cd-induced oxidative stress, thereby contributing to physiological and biochemical balance (Villiers et al. 2012; Karwel et al. 2020).

In response to energy loss caused by stress from Cd ions, the phytohormone 24-EBL stimulates the enzyme ribulose-1,5-bisphosphate carboxylase/oxygenase (RuBisCO), a key enzyme in the photosynthesis process (Kagale et al. 2007; Jiang et al. 2012; Nguyen et al. 2021). This enzyme is responsible for fixing carbon dioxide (CO₂) during the conversion of light energy into carbohydrates (Koch 1996; Li et al. 2005; Zhu et al. 2016; Yin et al. 2022). This additional regulation enables the plant to accumulate carbohydrate reserves that can later be used to meet energy demands during stress and recovery (Gill and Tuteja 2010; Zhao et al. 2017; Wang et al. 2017; Huque et al. 2021).

The roots of plants are the first line of contact with the environment and are therefore more susceptible to the absorption of heavy metals, such as Cd, present in the soil (Baranda et al. 2019). Additionally, roots contain a higher concentration of binding sites and ion transporters, which increases Cd uptake compared to leaves (Iori et al. 2017; Sabella et al. 2022). However, in extreme cases of toxicity, plants may strengthen their roots through lignification, converting meristematic cells into xylem cells and depositing lignin, which results from the synthesis and polymerization of phenolic monomers such as coniferaldehyde (Wang et al. 2019; Kobyletska et al. 2020; Yan et al. 2023).

This process provides mechanical resistance, improves water and nutrient conduction, and seals intercellular spaces, reducing exposure to Cd in the soil and increasing resistance to the metal (Muneer et al. 2012; Zhu 2016; Zulfiqar et al. 2022). However, despite its importance, lignification does not completely eliminate the stress caused by Cd (Xu et al. 2013; Balk et al. 2023). Plants also employ various intracellular purification strategies, such as using antioxidant enzymes to eliminate reactive oxygen species (ROS) and mitigate oxidative effects in root cells (Arora et al. 2010; Kwinta et al. 2019; Raza et al. 2022).

When it comes to endemic species of the Amazon biome, African mahogany (*Khaya grandifoliola*) accumulates Cd in its roots, while in the species Ucuúba (*Virola surinamensis*), under the influence of Cd, an increase in reducing sugars in the roots has been observed, promoting osmotic adjustment and tissue protection (Paiva et al. 2021; Júnior et al. 2021). Research conducted on *S. parahyba* var. *amazonicum* in unfavorable environments, in the presence of zinc (Zn) and with the use of silicon (Si) as a moderating element, demonstrated a reduction in nutritional deficiencies, highlighting the species as a phytostabilizer (Albuquerque et al. 2020). These characteristics underscore the physiological adaptations of Amazonian species in polluted environments.

### Changes in the photosynthetic pigments of *S. parahyba var. amazonicum (Huber ex Ducke) Barneby (PARICÁ)* seedlings under different concentrations of CdCl₂ and 24-EBL

At high concentrations, Cd ions can replace Mg in chlorophyll or Fe in ferredoxins (Eller and Brix 2015; Çikili et al 2016; Chen et al. 2019). Magnesium ions (Mg^2^⁺) play an essential role in light absorption within the chlorophyll molecule, particularly in its central porphyrin ring (Benavides et al. 2005; Hajjaoui et al. 2022). When Cd replaces Mg^2^⁺, photosynthesis efficiency is compromised, as Mg^2^⁺ is crucial for light absorption and electron transfer during this process (Song et al. 2019; Seregin et al. 2021).

High doses of Cd affect protein complexes that are essential in the electron transport chain during photosynthesis, inhibiting their activity and compromising the efficient transfer of electrons (Bajguz 2011; Sachdev et al. 2021). This adverse effect results in a decrease in ATP (adenosine triphosphate) and NADPH (nicotinamide adenine dinucleotide phosphate reduced), both crucial for the synthesis of glucose and other vital components of photosynthesis (Parmar et al. 2013; Gutsch et al 2018; Sabir et al. 2019). The exogenous application of 24-EBL has shown a significant role in improving tolerance to oxidative stress induced by Cd metal in plants (Jan et al. 2018). When sprayed on leaves, 24-EBL acts as a key modulator, activating intracellular antioxidant systems (Yu et al. 2017; Hasanuzzaman et al. 2017; Stirk et al. 2018). It induces the synthesis of antioxidant enzymes such as SOD, GPx, and CAT, which help neutralize the free radicals generated by Cd, thereby minimizing oxidative damage to biomolecules (Raza et al. 2013; Shah et al. 2019). Additionally, they promote the physiological maintenance of leaves, stimulating stomatal closure and reducing water loss through transpiration, thus helping preserve cellular membrane integrity and photosynthetic efficiency (Palliotti et al. 2015; Mohammad et al. 2020; Júnior et al 2022; Guo et al. 2023).

The excessive accumulation of Cd within plant cells promotes the formation of ROS, which are naturally present in plants but in minimal amounts under normal conditions (Simkin et al. 2019).

At the biochemical level, there is also an increase in the concentration of non-enzymatic antioxidant compounds, such as polyphenols and flavonoids, enhancing the plant’s ability to combat oxidative stress caused by Cd (Asami et al. 2005; Yadav et al. 2016; Emamverdian et al., 2020). To prevent oxidative stress, plants can diversify their strategies to combat reactive oxygen, such as the metabolism of *CAR* and *ACN* (Andrianos et al. 2016; Yao et al. 2022; Mulyaningsih et al. 2023). *CAR* and *ACN* play an essential antioxidant role during photosynthesis by neutralizing singlet oxygen activity (Mahalakshm, 2017; Enaru et al. 2021; Gupta 2023). They interact with lipid peroxides to interrupt the chain production of ROS, eliminate excited chlorophyll molecules to prevent the formation of singlet oxygen, and neutralize excess excited energy during the xanthophyll cycle (Behrens et al. 2019; Cerqueira et al. 2023).

### Fluctuation of CYP_*85*_*A*_*2*_ and *BZR1* genes in the aerial part and roots of *S. parahyba var*. *amazonicum (Huber ex Ducke) Barneby* (PARICÁ) subjected to different concentrations of CdCl₂ and 24-EBL

In the presence of high amounts of Cd ions in the aerial part, the formation of reactive oxygen species (ROS) occurs, affecting photosynthesis, stomatal conductance, the RuBisCO enzyme, and transpiration (Nogueira et al. 2019). In situations of physiological stress, *BZR1* orchestrates the expression of genes related to antioxidant enzymes (Khan et al. 2023). It operates by neutralizing ROS, such as SOD, which converts superoxide radicals into hydrogen peroxide (H_2_O_2_), while CAT decomposes peroxide (H₂O₂) into H₂O and O₂ (Huang et al. 2019). GPx uses reduced glutathione to reduce H₂O₂ into less harmful compounds (Salama et al. 2022). Ascorbate peroxidase (APX) neutralizes H_2_O_2_ using ascorbic acid as a cofactor (Alam et al. 2020). Glutathione reductase (GR) regenerates reduced glutathione, maintaining its antioxidant role (Cao et al. 2013; Santos et al. 2018). The combined action of these enzymes forms a coordinated system that protects cells from reactive oxygen species, preventing oxidative damage (Considine and Foyer 2014; Goncharuk and Zagoskina 2023).

In the case of the species *S. parahyba*, due to its epigeal characteristic, it initially releases the hypocotyl, pushing the cotyledons to the soil surface, followed by the protrusion of the radicle (Dutra et al. 2017; Moura et al 2019; Cunha et al 2020). This process results in roots, which are the last structures to undergo cellular differentiation and expansion of vascular tissues, resulting in higher levels of brassinosteroids (Arantes et al. 2020). *CYP*_*85*_*A*_*2*_, by influencing the synthesis of BR, triggers a cascade of events that positively impact cell division and organ formation (Gan et al. 2022; Zhang et al. 2024). BR, in turn, interacts in the cell nucleus with *BZR1*, regulating the expression of genes related to hormonal response and cell division (Burger and Chory 2019; Landi and Sharma 2021). Auxin production is strictly related to the interaction of the genes *CYP*_*85*_*A*_*2*_ and *BZR1*, modulating their levels to define plant architecture, determining the spacing and orientation of cells (Gruszka 2018; Nosak et al. 2021).

Plants exposed to high levels of Cd toxicity exhibit changes in their cells, and the CYP_85_A_2_ gene is activated, promoting the biosynthesis of phytoesteroids (24-EBL), plant hormones that help reduce the toxic effects of Cd (Mussig et al. 2002; Kim et al. 2005; Chakraborty et al. 2023). Under stress conditions in the cells, the phytohormone 24-EBL binds to its receptor on the cell membrane (Anwar et al. 2018a, b; Nolan et al. 2020). This triggers intracellular signaling of the *BZR1* gene, generating mRNA in the nucleus (Nam and Li 2002; Kim et al. 2010; Fang et al 2021; Zuo et al. 2022). This mRNA is translated into the *BZR1* protein by ribosomes in the cytoplasm (Ryu et al. 2007; Eremina et al. 2016; Riverola et al. 2019). The *BZR1* protein acts as a transcription factor, binding to DNA in the nucleus and directing genes associated with Cd tolerance, regulating their expression and the quantification of enzymes and proteins involved in cell homeostasis regulation (Li et al. 2016; Bruno et al. 2021; Kono and Yin 2020).

In addition, *BZR1* regulates the activity of genes related to the synthesis of phytochelatin proteins (Kim et al. 2019; Xian et al. 2020). These proteins have the ability to bind to Cd and other heavy metals, reducing their toxicity in plant cells (Durán et al. 2013). *BZR1* also aids in osmotic regulation by stimulating genes that encode compatible solutes, such as soluble sugars, which help maintain the osmotic balance of cells, preventing dehydration and maintaining cellular equilibrium (Oliveira et al. 2002; Fariduddin et al. 2014; Hewedy et al. 2022).

According to Nogueira et al. (2022), the species *S. amazonicum*, classified as a phytoextractor, accumulates Cd ions mainly in the roots, despite their presence in the aerial part. Studies indicate that the low activity of *CPY*_*85*_*A*_*2*_ and *BZR1* reduces the production of BR, limiting cell division and allowing the increase of genes related to lignification (Yu et al. 2004; Rovere et al. 2022; Li et al. 2023). Recent research highlights the essential role of genes related to the lignification of the cell wall in the roots for Cd absorption, transport, and tolerance (Li et al. 2021, 2022; Shangguan et al. 2023). However, this mechanism compromises the root contact surface, resulting in the shortening of the root system (Han et al. 2022).

### Variation of the CAD1 gene in the aerial part and roots of* S. parahyba *var.* amazonicum (Huber ex Ducke) Barneby (PARICÁ) *subjected to different concentrations *of CdCl*_*2*_* and 24-EBL*

The influence of *CAD1* in the root cells of plants in response to Cd concentration involves a complex genetic regulation mechanism (Couto 2016; Nounurai et al 2022). When the roots detect high concentrations of Cd in their environment, the activation of specific transcription factors occurs, which bind to the *CAD1* promoter (Holmes et al. 2021). This triggers the transcription of *CAD1*, resulting in the production of corresponding mRNA (Jiang et al 2023). This mRNA is then translated into *CAD1* proteins, which act as Cd transporters in the root cells (Ha et al 1999; Cazale and Clemens 2001; Thevenin et al. 2011). This molecular response helps plants cope with Cd stress by storing it in the vacuole and reducing damage throughout the plant. This is crucial for the survival of plants under exposure to the toxic metal (Howden et al. 1995; Eudes et al. 2006; Benedictis et al. 2018).

The application of 24-EBL can maximize the production of phytochelatins (PCs) in the root cells of plants, becoming crucial for the plant’s response to stress caused by heavy metals and other environmental pollutants (Reeves et al. 2018). According to Seregin and Kozhevnikova (2023), these small molecules are synthesized through a series of complex biochemical reactions that mainly occur in the cytoplasm of root cells. PCs are activated when the plant detects the presence of toxic metal ions, such as Cd or lead (Andresen et al. 2013; Uraguchi et al. 2021). This happens as part of the plant’s defense mechanism to protect against the excessive absorption of these harmful metals (Yuan et al 2008; Song et al. 2017; Kozhevnikova et al. 2020).

Firstly, 24-EBL positively influences the production of glutathione in the cells. It regulates the expression of genes involved in glutathione synthesis, such as γ-glutamylcysteine synthetase (γ-ECS) and glutathione synthetase (GS) (Wiszniewska et al. 2019). This results in an increase in intracellular glutathione levels, which is a crucial molecule for antioxidant defense and cellular detoxification (Chaudhuri et al. 2022).

Moreover, 24-EBL is also involved in the production of PCs. These phytochelatins are formed from glutathione residues and play a critical role in the uptake and sequestration of toxic metal ions in root cells (Corso et al. 2018; Kour et al. 2021).

In summary, the presence of Cd in plant cells can cause changes in their structure and function, involving morphological, biochemical, and physiological aspects (Angulo et al. 2021). Over time, plants have developed adaptive genetic mechanisms to cope with Cd and maintain their biological stability (Hou et al. 2023). 24-EBL, derived from cycloartenol, plays a crucial role by influencing the gene expression linked to Cd tolerance, affecting the transcription of DNA into mRNA (Anwar et al. 2018a, b; Rizvi et al. 2020). This gene regulation by 24-EBL can result in changes in the production of antioxidant enzymes, osmotic compounds, and others that help reduce the toxic effects of Cd (Sharma et al. 2023). The intracellular interaction between RNA, DNA, and phytohormones like 24-EBL is essential for the plant’s response to Cd toxicity and its adaptation to contaminated environments (Shu et al. 2016; Sharma et al. 2018; Tian et al. 2022). Although there is little understanding of how this hormone induces molecular tolerance mechanisms in Amazonian plant biodiversity, its role is extremely important for the survival of plants in territories polluted with heavy metals.

## Conclusion

In the context of this study, Cd^2^⁺ interfered with chlorophyll content (*Chl a*, *Chl b*, and *Chl a* + *b*) and seedling height of *Schizolobium parahyba* var. *amazonicum*, despite the application of 24-EBL (0, 20, and 40 nM). However, an increase in the *CAR* and *ACN* variables was observed, contributing to the reduction of oxidative stress. In parallel, the CAD1 gene acted restrictively up to 50 µM CdCl_2_ in both plant organs (leaves and roots), supporting the production of phytochelatins (PCs) for Cd^2^⁺ transport. Additionally, molecular analyses revealed an upregulation of the *CYP*_*85*_*A*_*2*_ and *BZR1* genes in the leaflets when combined with 40 nM 24-EBL, suggesting a significant role in energy conversion and the maintenance of cellular stability. These findings indicate the presence of protective mechanisms and genetic regulation that may extend biological responses against the harmful effects of CdCl_2_. Therefore, further in-depth studies on gene interactions modulated by 24-EBL in *S. parahyba* var. *amazonicum* are essential to elucidate the molecular and physiological mechanisms underlying homeostasis and species tolerance to Cd toxicity.

## Data Availability

The original contributions presented in this study are included in the article. Further inquiries can be directed to the corresponding author.

## Abbreviations

ACN: Anthocyanins
APX: Ascorbate peroxidase
BR: Brassinosteroids
BRZ1: Brassinazole-resistant 1
CdCl_2_: Cadmium chloride
CAD1: Phytochelatin synthase 1
Chl a: Chlorophyll a
Chl b: Chlorophyll b
Chl (a + b): Total chlorophyll
Clo a: Chlorophyll a
Clo b: Chlorophyll b
Clo a + b: Total chlorophyll
CAR: Carotenoids
CPY85A2: Cytochrome85A2
CAT: Catalase
DNA: Deoxyribonucleic acid
GR: Glutathione reductase
H_2_O_2_: Hydrogen peroxide
mRNA: Messenger ribonucleic acid
PRA%: Percentage of loss and gain
qPCR-TR: Real-time polymerase chain reaction
RNA: Ribonucleic acid
SOD: Superoxide dismutase
24-EBL: 24-Epibrassinolide
